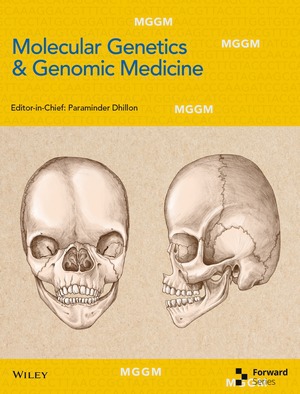# Cover Image

**DOI:** 10.1002/mgg3.70227

**Published:** 2026-04-28

**Authors:** Dominique L. Assing, Danielle E. Jolly, Sarah Gluschitz, Beverly Nelson, Dong Li, Elizabeth J. Bhoj, Tomoki T. Nomakuchi, Andrew K. Sobering

## Abstract

The cover image is based on the article *Clinical and Neurodevelopmental Course in a Case of EFNB1‐Related Craniofrontonasal Syndrome With Unrepaired Craniosynostosis* by Dominique L Assing et al., https://doi.org/10.1002/mgg3.70216.